# A cohort study on acute ocular motility disorders in pediatric emergency department

**DOI:** 10.1186/s13052-018-0502-0

**Published:** 2018-05-29

**Authors:** Umberto Raucci, Pasquale Parisi, Nicola Vanacore, Valentina Ferro, Giacomo Garone, Federica Sancetta, Sergio Petroni, Stefano Pro, Rossella Rossi, Antonino Reale, Nicola Pirozzi

**Affiliations:** 1 Pediatric Emergency Department, Bambino Gesù Children’s Hospital, IRCCS, Piazza S. Onofrio 4, Rome, Italy; 2 Chair of Pediatrics, NESMOS Department, Faculty of Medicine and Psychology, Sapienza University, c/o Sant’Andrea Hospital, Rome, Italy; 3 National Centre for Epidemiology, Surveillance, and Health Promotion, National Institute of Health, Rome, Italy; 4 University Hospital Pediatric Department, Bambino Gesù Children’s Hospital, IRCCS Tor Vergata University, Rome, Italy; 5 Pediatric Division, SS Giovanni and Paolo Hospital, Venice, Italy; 6 Ophthalmology Department, Bambino Gesù Children’s Hospital, IRCCS, Rome, Italy; 7 Neurology Unit, Department of Neurosciences, Bambino Gesù Children’s Hospital, IRCCS, Rome, Italy

**Keywords:** Child, Emergency department, Acute strabismus, Ptosis, Pupillar disorder, Red flags, Emergency department

## Abstract

**Background:**

Acute ocular motility disorders (OMDs) in children admitted to Emergency Department (ED) represents a not so rare condition with a wide spectrum of different etiologies. The emergency physician must be skilled in rapidly identifying patients with potentially life threatening (LT) forms, requiring further diagnostic procedures. The aim of the study was to assess characteristics of children with acute Ocular Motility Disorders (OMDs), and to identify “red flags” for recognition of underlying life-threatening (LT) conditions.

**Methods:**

A retrospective cohort study evaluated children (2 months-17 years) admitted to a tertiary Emergency Department in 2009–2014. A subgroup analysis was performed comparing children with and without LT conditions.

**Results:**

Of 192 visits for OMDs, the isolated strabismus occurred most frequently (55.6%), followed by pupil disorders (31.8%), ptosis (5.2%) and combined OMDs (11.5%). The majority of acute OMDs involved no underlying LT conditions (*n* = 136) and most of them were infants or toddlers (50%). In a multivariable analysis, LT conditions included especially children over 6 years of age, increasing the odds ratio by 2% for each months of age (*p* = 0.009). LT etiologies were 16 times more likely in combined OMDs (*p* = 0.018), were over 13 times more likely to report associated extra-ocular signs/symptoms (*p* = 0.017) and over 50 times more likely to report co-morbidity (*p* = 0.017).

**Conclusion:**

OMDs are not an uncommon presentation at ED. Although most of them involve non-LT conditions, the ED physician should consider potential “red flags” for appropriate management of children such as age > 6 years, combined OMDs, extra-ocular symptoms and co-morbidity.

## Background

Acute ocular motility disorders (OMDs) are a frequent reason for admission to an outpatient general hospital or to a pediatric Emergency Department (ED). The clinical manifestations of such disorders are represented by strabismus, ptosis, mydriasis (unilateral or bilateral) and miosis, with isolated or associated presentations. In fact, these disorders present with a wide variety of clinical pictures and underlie different etiologies, both congenital and acquired (head injury, infectious diseases, neurological and neoplastic diseases, toxic substances (systemically administered or topically instilled), vascular and autoimmune diseases), with different incidence rates compared to adults [1, 2]. So, the emergency physician must be skilled in rapidly identifying those patients requiring further diagnostic procedures such as neuroradiological investigations, which could be superfluous and potentially dangerous in the developmental age.

To our knowledge, data on presentation and management of OMDs in the pediatric population, especially in ED, are lacking. Previous literature studies report limited knowledge, being mainly represented by case reports or reviews focusing only on specific ocular deficit. Our study analyzed by medical charts review 6 years of experience in the ED of a tertiary pediatric hospital, with the aim to investigate etiology and management of OMDs. Specifically, the study planned to describe general characteristics, etiology and health care resources utilization, trying to identify “red flags” significantly correlated with potential life threatening (LT) conditions and to address future research.

## Methods

After obtaining approval from the institutional ethics committee, we conducted a retrospective cohort study of patients, aged between 2 months and 17 years, presenting with a primary complaint of acute OMDs to the ED of the Bambino Gesù Children’s Hospital in Rome, between January 2009 and December 2014. There is an ongoing scientific collaboration and an agreement between the ED of the Bambino Gesù Children’s Hospital and the Post-Graduate Schools in Pediatrics of both Tor Vergata University of Rome and the Chair of Pediatrics, Faculty of Medicine and Psychology of Sapienza, University of Rome. Exclusion criteria were represented by neonatal age and patients in whom the diagnosis of OMDs was already known.

The following data were extracted from each medical record: age, gender, triage code, time of onset, medical history, ocular and extra-ocular signs/symptoms (systemic and/or neurological), physical examination findings, specialist consultations, imaging techniques such as computed tomography (CT) scan and magnetic resonance imaging (MRI), final diagnosis, hospital admission and length of stay, where applicable.

We decided to stratify our sample according to age in three groups: 2–36 months (infant/toddler), 37–72 months (pre-schooler children) and 73–214 months (schooler- teenager).

The following codes were used to describe the patient condition at the time of triage: red or immediate (need to be seen immediately), yellow or very urgent, with high priority (need to be seen in less than 15 min), green or urgent (need to be seen in 60–120 min), white or non-urgent (need to be seen after previous triage codes). OMDs were divided into four subgroups: isolated pupillar disorders, isolated strabismus, isolated ptosis and combined ocular disorders.

According to ED discharge diagnosis, patients were classified in two groups basing on the condition causing the OMD: potentially life threatening (LT) diseases (metabolic diseases, cerebrovascular diseases, brain tumors, cerebral infections, pseudotumor cerebri, demyelinating diseases, brain injuries, myasthenia gravis, Bernard-Horner syndrome) and non-LT (NLT) diseases (transient condition, pharmacologic/toxic causes, seizure, ocular disease, cranial nerve deficit, migraine, movement disorder). Then, we compared children with a LT disease with the other children (NLT).

### Statistical analysis

We described the clinical and demographic features of all the patients enrolled, providing details of the overall sample as well as of each of the two subgroups (patients with and without LT conditions). The two groups were compared by means of the Chi-square test for categorical variables, and Student’s t test for continuous variables, after reviewing for appropriateness. We applied a logistic regression analysis model to assess the predictive variables associated with a diagnosis of LT conditions. Adjusted odds ratios (OR) and 95% confidence intervals (CI) were used as measures of effect. The statistical significance was set at *p* < 0.05 and SPSS software (version 22.0) was used to perform all the statistical analysis.

## Results

### Descriptive analysis of the overall study population

During the 6-year study, among a total of 304,224 children admitted to our ED, 192 subjects presented with acute OMDs, at a rate of 0.6 visits per 1000 children. Isolated strabismus occurred most frequently (*n* = 99; 51.6%) followed by isolated pupillary disorders (*n* = 61; 31.8%), ptosis (*n* = 10; 5.2%) and combined OMDs (*n* = 22; 11.5%) (Table 1).

**Table 1:** Characteristics of the overall population study and of four considered ocular motility disorders

Characteristics	Total *n* = 192 (%)	Isolated Pupillar Disorders *n* = 61 (31.8%)	Isolated Strabismus *n* = 99 (51.6%)	Isolated Ptosis *n* = 10 (5.2%)	Combined Ocular Disorders *n* = 22 (11.5%)
Sex					
Female	87 (45.3%)	28 (45.9%)	43 (43.4%)	4 (40%)	12 (54.5%)
Male	105 (54.7%)	33 (54.1%)	56 (56.6%)	6 (60%)	10 (45.5%)
Age (months) (mean ± SD)	63.84 ± 52.02	60.51 ± 60	60.34 ± 44.7	102.4 ± 54.32	71.32 ± 54.8
Age group					
Infant –Toddler (2–36 months)	78 (40.6%)	31 (50.8%)	40 (40.4%)	2 (20%)	5 (22.7%)
Preschooler (37–72 months)	45 (23.4%)	10 (16.4%)	26 (26.3%)	1 (10%)	9 (36.4%)
Schooler-Teenager (73–214 months)	69 (35.9%)	20 (32.8%)	33 (33.3%)	7 (70%)	9 (40.9%)
Triage					
White	6 (3.1%)	2 (3.3%)	2 (2%)	1 (10%)	1 (4.5%)
Green	88 (45.8%)	23 (37.7%)	51 (51.5%)	4 (40%)	10 (45.5%)
Yellow	93 (48.4%)	31 (50.8%)	46 (46.5%)	5 (50%)	11 (50%)
Red	5 (2.6%)	5 (8.2%)	0	0	0
Clinical Onset					
Within 24 h	92 (47.9%)	41 (67.2%)	39 (39.4%)	3 (30%)	9 (40.9%)
Within 72 h	28 (14.6%)	8 (13.1%)	16 (16.2%)	2 (20%)	2 (9.1%)
Over 72 h	72 (37.5%)	12 (19.7%)	44 (44.4%)	5 (50%)	11 (50%)
Systemic Symptoms	92 (47.9%)	27 (44.3%)	47 (47.5%)	9 (90%)	9 (40.9%)
Specialist Consultation at ED	167 (87%)	45 (73.8%)	94 (94.9%)	8 (80%)	20 (90.9%)
NeuroImaging	118 (61.5%)	22 (36.1%)	69 (69.7%)	9 (90%)	18 (81.8%)
CT Scan	92 (47.9%)	21 (34.4%)	50 (50.5%)	7 (70%)	14 (63.6%)
MRI	83 (43.2%)	10 (16.4%)	50 (50.5%)	8 (80%)	15 (68.2%)
Outcome					
Discharged	71 (37%)	30 (49.2%)	36 (36.4%)	2 (20%)	3 (13.6%)
Hospitalized	121 (63%)	31 (50.8%)	63 (63.6%)	8 (80%)	19 (86.4%)
Lenght of Hospital Stay (mean ± SD)	10.42 ± 12.8	9.21 ± 14	9.19 ± 10.3	13 ± 6.1	16.8 ± 18.41
Life-Threatening Conditions	56 (29.2%)	10 (16.4%)	27 (27.3%)	6 (60%)	13 (59.1%)

*SD* Standard Deviation, *ED* Emergency Department, *CT* Computed Tomography, *MRI* Magnetic Resonance Imaging

Our population comprised of 87 females (45.3%) and 105 males (54.7%), (M/F ratio 1.2). Males prevailed in all OMDs group except among patients with combined OMDs (Table 1).

Children were aged from 2 to 214 months (mean age 63.34 ± 52.06 months, median 45.5) (Table 1). Most children were aged between 2 and 36 months (*n* = 78; 40.6%), (Table 1). In this age group included pupil disorders and strabismus prevailed, whereas children aged over 6 years old presented more frequently with ptosis and combined OMDs (Table 1).

The OMD onset occurred more frequently within 24 h before ED visit, with pupil disorders showing an onset in less than 24 h in over two thirds of the cases, and the other disorders appearing more frequently after 72 h (Table 1). On admission, the triage code was mainly yellow and green (Table 1). Ninety-two children complained of extra-ocular symptoms, involving mostly children with ptosis followed by strabismus (Table 1). Twenty-seven patients reported co-morbidities (14.1%).

Children on current medical treatment accounted for 7.3% of the cohort, more frequently among patients with isolated pupillary disorders (18% in this group); the accidental exposure to substances or medications was found only in children with pupillary disorders, accounting for 6,8% (13 patients).

In many cases, a specialist consultation was needed (*n* = 167; 87%) (Table 1) with neurological and ophthalmological consultations required most frequently (respectively in 51.6 and 69.8% of the patients). Less frequently, consultations by neurosurgeon (*n* = 11; 5.7%), intensive care physician (n = 11; 5.7%) or maxillofacial surgeon (*n* = 3; 1.6%) were requested. Neurologist referral was performed mostly in children with ptosis (*n* = 7; 70%), while ophthalmologist consultation was required mainly for children with ptosis (*n* = 5; 50%) and pupillary disorders (*n* = 29; 47.5%).

Neuroimaging was performed in 118 children (61.5%), mainly in patients with pupillary disorders (90%) and combined OMDs (81.8%) (Table 1), CT and MRI having been performed in 47.9 and 43.2% of the patients, respectively (Table 1). Both CT and MRI were more frequently performed in patients with ptosis (respectively 70 and 80%), followed by children with combined OMDs (63.6 and 68.2%, respectively, Table 1). Among children admitted for OMDs, 63% were hospitalized, with an average length of stay of 10.42 days. Patients with ptosis and combined OMDs were more frequently hospitalized (80 and 86.5%, respectively), with a longer hospital stay compared to the other OMDs (Table 1).

### LT etiologies: Clinical characteristics and diagnostic findings

OMDs underlied a LT conditions in 56 children (29.2% of the entire sample), more frequently among children with ptosis and combined OMDs (Table 1).

**Fig. 1:**
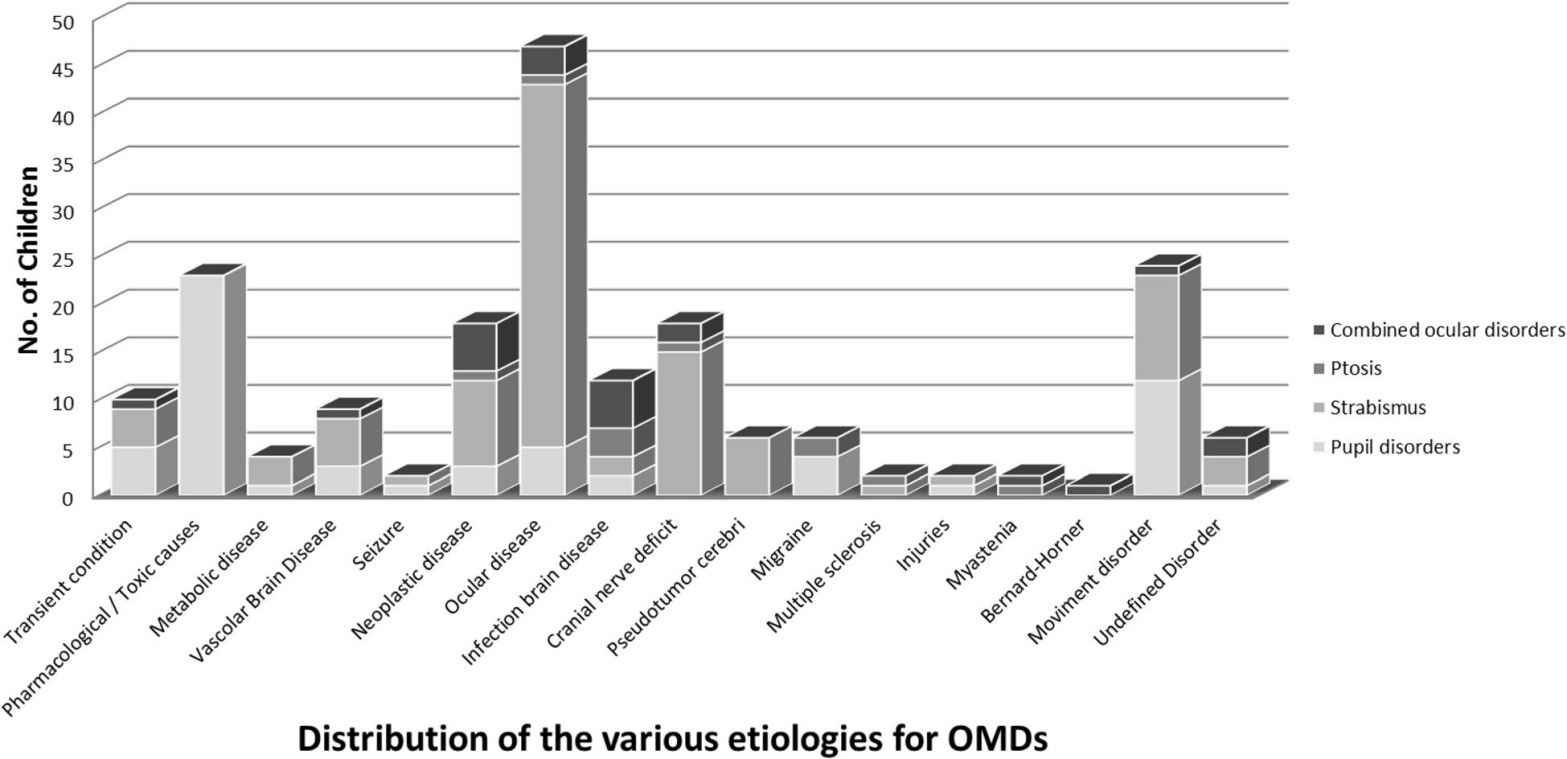
Distribution of the various etiologies for acute ocular motility disorders; OMDs: Ocular Motility Disorders

The distribution of the various etiologies for acute OMDs is reported in Fig. 1. There was a significant difference in the frequency of LT conditions in the different age groups (Table 2). On triage, a yellow code was more frequently assigned to children with LT conditions (*p* = 0.008) (Table 2). In the LT group, symptoms onset was more frequently as far as 72 h before the time of the examination. In the other group, the onset was more commonly within the 24 h preceding the ED visit (*p* = 0.003) (Table 2). Extra-ocular manifestations were significantly more common in the LT group (*p* = 0.001) and LT conditions were significantly associated with combined OMDs (*p* = 0.002) (Table 2). The co-morbidities were more frequently seen in children with LT etiologies (*p* = 0.002) (Table 2).

**Table 2:** Clinical and demographic characteristics of the two subgroups (Non-Life Threatening and Life Threatening conditions)

Characteristics	Non-Life Threatening n = 136	Life Threatening n = 56	*p* value
Sex			NS
Female	59 (43.4%)	28 (50%)	
Male	77 (56.6%)	28 (50%)	
Age group			0.001
Infant -Toddler	68 (50%)	10 (17.9%)	
Preschooler	28 (20.6%)	17 (30.4%)	
Schooler-Teenager	40 (29.4%)	29 (51.8%)	
Age (months)(mean ± SD)	52.8 ± 47	90.66 ± 54.2	0.001
Triage			0.008
White	6 (4.4%)	0	
Green	71 (52.2%)	17 (30.4%)	
Yellow	56 (41.2%)	37 (66.1%)	
Red	3 (2.2%)	2 (3.6%)	
Clinical Onset			0.003
Within 24 h	76 (55.9%)	16 (28.6%)	
Within 72 h	17 (12.5%)	11 (19.6%)	
Over72 hours	43 (31.6%)	29 (51.8%)	
SystemicSymptoms			0.001
No	92 (67.6%)	8 (14.3%)	
Yes	44 (32.4%)	48 (85.7%)	
Ocular Disorder			0.002
One disorder	127 (93.4%)	43 (76.8%)	
More one disorder	9 (6.6%)	13 (23.2%)	
Concomitant Disease			0.002
No	124 (91.2%)	41 (73.2%)	
Yes	12 (8.8%)	15 (26.8%)	
During Medical Treatment			0.048
No	123 (90.%)	55 (98.2%)	
Yes	13 (9.6%)	1 (1.8%)	
Specialist Consultation at ED			NS.
No	18 (13.2%)	7 (12.6%)	
Yes	118 (86.8%)	49 (87.6%)	
Neurologic Consultation			NS
No	68 (50%)	25 (44.6%)	
Yes	68 (50%)	31 (55.4%)	
Ophthalmologic Consultation			0.058
No	36 (26.5%)	22 (39.3%)	
Yes	100 (73.5%)	34 (60.7%)	
Neurosurgical Consultation			0.001
No	135 (99.3%)	46 (82.1%)	
Yes	1 (0.7%)	10 (17.9%)	
Anesthesiologic Consultation			NS
No	129 (94.9%)	52 (92.9%)	
Yes	7 (5.1%)	4 (7.1%)	
Maxillo-Facial Surgery Consultation			NS
No	134 (98.5%)	55 (98.2%)	
Yes	2 (1.5%)	1 (1.8%)	
Outcome			0.001
Discharged	71 (52.2%)	0	
Hospitalized	65 (47.8%)	56 (100%)	
Lenght of Hospital Stay(mean ± SD)	4.45 ± 4.7	17.59 ± 15.32	0.001
Neuro Imaging			0.001
No	74 (54.4%)	0	
Yes	62 (45.6%)	56 (100%)	
CT Scan			0.001
No	90 (66.2%)	10 (17.9%)	
Yes	46 (33.8%)	46 (82.1%)	
MRI			0.001
No	99 (72.8%)	10 (17.9%)	
Yes	37 (27.2%)	46 (82.1%)	

*SD* Standard Deviation, *ED* Emergency Department, *CT* Computed Tomography, *MRI* Magnetic Resonance Imaging

With regards to pharmacological aspects, children on current treatment appeared mainly in the NLT group (*p* = 0.048) (Table 2).

Among specialist consultations, only neurosurgeon evaluation was more frequently requested in the LT group (p = 0.001) (Table 2). All children with LT conditions underwent neuroimaging (*n* = 56; 100%), versus 45.6% (62 patients) in the other group (*p* = 0.001).

All patients with LT conditions and 65 patients (47.8%) with NLT conditions were hospitalized. The hospital stay was significantly longer in the LT group (*p* = 0.001) (Table 2).

### Logistic regression

Regarding the children’s age, the odds that an older child had LT conditions increased by 2% for each month of age (OR = 1.02; CI 95%: 1.005–1. 036; *p* = 0.009) (Table 3).

**Table 3:** A regression logistic model of risk of Life Threatening conditions in children with Ocular Motility Disorders

Variable	B	SE	Wald	DF	Sig.	OR	Cl 95%	
							Lower	Upper
Age (months)	0.020	0.008	6.744	1	0.009	1.02	1.005	1.036
Triage Code (yellow vs others)	0.410	0.707	0.336	1	0.562	1.51	0.377	6.019
Clinical Onset within 24 h			4.731	2	0.094	1.00		
Clinical Onset within 72 h	2.395	1.378	3.020	1	0.082	11.00	0.736	163.414
Clinical Onset over 72 h	1.486	0.796	3.486	1	0.062	4.42	0.929	21.011
Ocular Disorder (combined vs isolated)	0.777	1.170	5.632	1	0.018	16.07	1.622	159.252
Systemic Symptoms	2.575	1.079	5.697	1	0.017	13.13	1.585	108.811
Concomitant Disease	3.925	1.647	5.677	1	0.017	50.66	2.006	1278.854
Children on Medical Treatment	−2.585	2.213	1.364	1	0.243	0.08	0.001	5.769
Ophthalmologic Consultation		−0.470	0.773	0.369	1	0.54	0.625	0.137
Neurosurgical Consultation	2.568	1.445	3.156	1	0.076	13.04	0.767	221.643
Lenght of Hospital Staying (days)	0.169	0.060	8.009	1	0.005	1.18	1.053	1.331
CT Scan	2.052	0.855	5.760	1	0.016	7.78	1.457	41.575
MRI	0.465	0.830	0.314	1	0.575	1.60	0.313	8.093
Constant	−8.473	1.858	20.795	1	0.000			

B: Coefficient (T-Statistics); *SE* Standard Error, *Wald* Test of Wald, *DF* Degree of Freedom, *Sig* Significance, *OR* Odds Ratio, *CI 95%* Confidential Interval 95%, *CT* Computed Tomography, *MRI* Magnetic Resonance Imaging

Considering the clinical subgroups, we reported that LT conditions were 16 times more frequent in combined OMDs (OR:16.07; CI 95%:1.622–159,25; *p* = 0.018) (Table 3).

Children with LT conditions were over 13 times more likely to report associated extra-ocular signs or symptoms (OR: 13.13; CI 95%: 1.585–108.811; *p* = 0.017) (Table 3).

Regarding clinical history, children of the LT group was 50 times more likely to report co-morbidities (OR:50.66; CI 95%:2.006–1278,85; p = 0.017) (Table 3). For hospitalized LT children, the length of stay was increased by 18% for each day (OR: 1.18; CI 95%: 1.053–1.331; *p* = 0.005). In relation to diagnostic investigations, children with LT conditions were over 7 times more likely to undergo to a CT (OR: 7.78; CI 95%: 1.457–41.575) (*p* = 0.016) (Tables 3 and 4).

**Table 4:** Red Flags for Ocular Motility Disorders in Emergency Department

- Combined Ocular Motility Disorders - Extraocular manifestation (i.e papilledema, headache, nausea or vomiting, weight loss) - Associated neurological signs and symptoms - Presence of co-morbidities - Ptosis - Age > 6 years - Strabismus also if isolated in subject with larger esodeviation at distance, recurrence of acute acquired concomitant esotropia	

## Discussion

OMDs are a not rare condition in ED, with a wide spectrum of different underlying causes. One of the strength-points in our study is the large sample of enrolled children, thus providing reliable epidemiological data from the ED of a Tertiary Pediatric Hospital, showing a rate of 0.6 visits per 1000 visits.

ED physicians must be skilled in rapidly identifying the few patients with potentially LT conditions of OMDs, requiring further diagnostic investigations such as neuro-radiologic investigations, that should be performed only if needed. However, differential diagnosis is challenging, and this may lead to excessive healthcare spending and unnecessary testing and treatment even for not severe conditions [3].

In our sample, most of the included patients were infants and toddlers and most of the admissions involved children who did not have underlying LT diseases (Table 1).

The leading cause of admission to our ED was isolated strabismus (55.6%) and the principal underlying cause were ocular disorders (38.4%), as described in the literature [4, 5]. Nevertheless, in our study, ocular nerve neuritis represented a common condition (15.2%), not reported in previous literature case series [4, 5].

The second cause of presentation was isolated pupillar disorders, accounting for 31.8% of the cohort. The foremost cause was toxic effects (37.7%) of drugs or other substances, given orally or topically (belladona, scopolamina, datura species and direct exposure to nebulized anticholinergic agents such as ipratropium). In case of topical exposure to anticholinergic agents, the pupil changes usually resolve in 24–48 h. Pilocarpine 1% test would confirm the pharmacologic effect [6]. For this reason, pediatricians should inquire about possible drug and toxic exposures and consider this diagnosis, especially in well-appearing patients, to avoid unnecessary and potentially dangerous diagnostic investigations [6].

Migraine diagnosis was estimated in 66.7% of pupillary disorders (Fig. 1), particularly in mydriasis (70.7%). The role of pupillary disorders in migraine patients is not completely understood [7, 8]. The association between migraine and monolateral or bilateral “tonic pupil” [9, 10] might be caused by hyperactivity of the sympathetic nervous system or hypoactivity of the parasympathetic system [8].

Combined OMDs occurred in 11.5% of patients, followed by isolated ptosis in 5.2% of children. In our study, the leading diagnosis of combined OMDs were LT conditions (59.1%), mainly neoplastic (22.7%) and infectious (22%) diseases. Combined OMDs such as ptosis with anisocoria in a child may be suggestive of Bernard-Horner syndrome, whose etiologies can include birth trauma, tumors such as neuroblastoma or benign neck masses [11]. In the case of third nerve palsy, combined OMDs are more easily recognized [4, 12]. Except for the first cause of third nerve palsy that is congenital (33–38%), the other causes are all acquired LT conditions: trauma (28–32%), tumors (22–11%), vascular (11%), meningitis (6%) [4]. Myasthenia Gravis, infrequently encountered in pediatric clinical practice, occurred in 4.5% of our children with combined OMDs in which ptosis was accompanied by strabismus.

In our study, isolated ptosis was determined mainly by LT conditions, such as brain infections. Of note, ophthalmoplegic migraine diagnosis was made in two children with ptosis; its recognition may save patients from unnecessary tests and interventions [13, 14] In these cases, it can be crucial to investigate on the presence of positive family history of idiopathic headache.

With the aim to identify clinical features predictive of LT conditions, we firstly highlight that children with LT disorders are more frequently older than 6 years of age. According to the logistic regression model, the odds that an older child had a LT conditions increased by 2% for each month of age. These data might be correlated to a higher frequency of acquired OMDs causes, occurring mostly over 6 years of age, respect to congenital causes, predominantly occurring in younger children. Our findings differ from previous studies in which a younger age at diagnosis was described [15–18], probably due to a higher frequency of congenital ptosis in these series compared to our study, where acquired conditions predominate. Similarly, the combined OMDs are more commonly associated with LT conditions (59.1%), involving especially the schooler-adolescent age group (40.9%). Nevertheless, we should consider that the label “congenital” does not always mean benign, as in neuroblastoma-related Bernard-Horner syndrome [19].

Secondly, in our study, the LT conditions were significantly associated with combined OMDs; in fact, the logistic regression reported that patients with LT etiologies were 16 times more likely to present more than a single OMD.

Nevertheless, ED physicians should consider that an isolated acute OMDs might be insidious as well. In fact, even if in our study isolated strabismus was associated with LT etiologies in a minority of cases, it should be kept in mind that the acute onset of esotropia can be due to a wide range of severe intracranial diseases [20]. In some cases, strabismus may represent the initial sign of a neurologic dysfunction [21]. With this regard, the “red flags” suggesting intracranial pathology are: larger esodeviation at distance, recurrence of acute acquired concomitant esotropia, neurological signs and older age at onset (> 6 years) [22]. In particular, in childhood, differently from adults, the acute onset of an esotropia is frequently associated with an underlying central nervous system disorder [23].

In the case of benign episodic mydriasis, with no other accompanying symptoms, with short-term episodes and in absence of abnormalities on neurological examination, imaging tests are not recommended.^8^ In our study the isolated pupillary disorders were associated with LT etiologies in only 16.4%. Accordingly, it is mandatory to search for red flags such as neurological signs or symptoms. In particular, the mydriasis associated with headaches should always alert the clinician to possibilities of serious potential LT conditions [24].

Similarly, acute-onset ptosis, that in our study is associated with life-threatening causes in 60% of the patients, may underlie serious etiologies, such as neuroblastoma, myasthenia gravis or muscular dystrophy, even presenting as an isolated finding [25, 26].

Our study demonstrates that the extra-ocular manifestations, such as papilledema, headache or nausea, weight loss and vomiting, were significantly associated with LT conditions; in addition, patients with LT conditions were 50 times more likely to report a comorbidity.

Given the broad spectrum of OMDs causes, if a certain benign diagnosis (i.e. ipratropium local exposure) cannot be made, in absence of red flags for a severe underlying etiology, ophthalmologic and neurologic consultations should be warranted before proceeding to further investigation, In fact, growing CT utilization at ED has been reported also in pediatric age; probably reflecting the increased availability of CT, the improvements in CT diagnostic capabilities, and an increased need of physicians and patients for diagnostic certainty [27]. Parallelly, a rise in CT use is associated with increased health care expenditures, increased hospitalization length, and increased exposure to ionizing radiation [28].

Conclusions of our work can be limited by the exposure to some confounding factors, as tends to be inevitable in any retrospective study design. In fact, some important and relevant details may not have been documented in clinical records. In addition, this is a single-center study performed at an academic tertiary Hospital and the frequency of some conditions could be overestimated at this care level.

## Conclusion

We can state that acute OMDs in children admitted to ED represents a not so rare condition with a wide spectrum of different etiologies. The leading clinical presentations in our sample was isolated strabismus, followed by isolated pupil disorders, ptosis and combined OMDs. Most of the admissions to ED for OMDs involved children who did not have underlying LT conditions and most of them were infants or toddlers who could be referred directly as outpatients to a pediatric ophthalmologist. Conversely, we would like to stress that children with LT conditions included especially children over 6 years of age. The risk of LT events was significantly higher in children with combined OMDs and/or associated extra-ocular signs/symptoms, with comorbidity in their clinical history. Obviously, further prospective studies are needed to improve OMDs management in pediatric ED.

## Abbreviations

CI: Confidence Interval
CT: Computed Tomography
ED: Emergency Department
LT: Life Threatening
MRI: Magnetic Resonance Imaging
NLT: Non-Life Threatening
OMDs: Ocular Motility Disorders
OR: Odd Ratio
SD: Standard Deviation
